# Lighting direction and visual field modulate perceived intensity of illumination

**DOI:** 10.3389/fpsyg.2013.00983

**Published:** 2013-12-24

**Authors:** Mark E. McCourt, Barbara Blakeslee, Ganesh Padmanabhan

**Affiliations:** Department of Psychology, Center for Visual and Cognitive Neuroscience, North Dakota State UniversityFargo, ND, USA

**Keywords:** brightness, perceived illumination, light-from-above bias, light-from-left bias, pseudoneglect, allocentric, egocentric, spatial attention

## Abstract

When interpreting object shape from shading the visual system exhibits a strong bias that illumination comes from above and slightly from the left. We asked whether such biases in the perceived direction of illumination might also influence its perceived intensity. Arrays of nine cubes were stereoscopically rendered where individual cubes varied in their 3D pose, but possessed identical triplets of visible faces. Arrays were virtually illuminated from one of four directions: Above-Left, Above-Right, Below-Left, and Below-Right (±24.4° azimuth; ±90° elevation). Illumination intensity possessed 15 levels, resulting in mean cube array luminances ranging from 1.31–3.45 cd/m^2^. A “reference” array was consistently illuminated from Above-Left at mid-intensity (mean array luminance = 2.38 cd/m^2^). The reference array's illumination was compared to that of matching arrays which were illuminated from all four directions at all intensities. Reference and matching arrays appeared in the left and right visual field, respectively, or vice versa. Subjects judged which cube array appeared to be under more intense illumination. Using the method of constant stimuli we determined the illumination level of matching arrays required to establish subjective equality with the reference array as a function of matching cube visual field, illumination elevation, and illumination azimuth. Cube arrays appeared significantly more intensely illuminated when they were situated in the left visual field (*p* = 0.017), and when they were illuminated from below (*p* = 0.001), and from the left (*p* = 0.001). An interaction of modest strength was that the effect of illumination azimuth was greater for matching arrays situated in the left visual field (*p* = 0.042). We propose that objects lit from below appear more intensely illuminated than identical objects lit from above due to long-term adaptation to downward lighting. The amplification of perceived intensity of illumination for stimuli situated in the left visual field and lit from the left is best explained by tonic egocentric and allocentric leftward attentional biases, respectively.

## Background

When interpreting the shape of ambiguous 3D surfaces the visual system exhibits a bias that directional illumination is mostly from above (Ramachandran, 1988; Sun and Perona, 1996a,b, 1998; Mamassian and Goutcher, 2001; Stone et al., 2009; de Montalembert et al., 2010; Morgenstern et al., 2011; Schofield et al., 2011; Andrews et al., 2013) and slightly from the left (Sun and Perona, 1998; Mamassian and Goutcher, 2001; Mamassian et al., 2003; McManus et al., 2004; Thomas et al., 2008; de Montalembert et al., 2010; Andrews et al., 2013). The light-from-above bias is well illustrated by experiments demonstrating that discs with top-dark luminance gradients are seen as concavities while those with top-bright luminance gradients are perceived as convexities (Ramachandran, 1988).

The light-from-above bias has also been invoked to explain an interesting visual search asymmetry, which is that visual search is efficient for targets lit from below amidst top-lit distractors, but is effortful for top-lit targets amidst bottom-lit distractors (Enns and Rensink, 1990; Sun and Perona, 1996a,b, 1998; but see Ostrovsky et al., 2005). Search is typically efficient for items distinguished from distractors by a single feature. These results imply that the attribute of being lit from below is a sufficiently uncommon stimulus attribute to be afforded status as a featural cue.

Another potentially related phenomenon is the finding that discs possessing top-dark luminance gradients, which are seen as concavities due to the light-from-above assumption, appear to possess higher contrast than identical top-bright discs which are seen as convexities (Chacon, 2004). Although the visual system exhibits a bias to assume convexity (Sun and Perona, 1997; Langer and Bulthoff, 2001; Champion and Adams, 2007), the light-from-above bias outweighs the convexity bias for simple disc stimuli. The mechanism resulting in the higher contrast appearance for the top-dark luminance gradients, however, remains unclear.

The bias that illumination is slightly from the left has been explained as attentional in origin. A large number of studies have shown that many object properties (e.g., brightness, numerosity, size) are exaggerated when they are situated in the left vs. right visual field (Bowers and Heilman, 1980; McCourt and Jewell, 1999; Nicholls et al., 1999; Jewell and McCourt, 2000; McCourt and Garlinghouse, 2000; Foxe et al., 2003; Charles et al., 2007). The commonly accepted explanation for this phenomenon, called pseudoneglect, is that it arises as a corollary of the right hemisphere's specialization for the deployment of visuospatial attention and the resultant prepotent vector of attention directed contralaterally into left hemispace (Heilman and Van Den Abell, 1979; de Schotten et al., 2011). Pseudoneglect has been demonstrated to occur in both space-based (egocentric) and object-based (allocentric) reference frames (Reuter-Lorenz et al., 1996; Nicholls et al., 2004; Orr and Nicholls, 2005; Pia et al., 2010).

### Purpose

Here we investigate the relationship between the visual and attentional effects of lighting direction within the same experiment by asking whether the direction of lighting of arrays of randomly posed stereoscopically rendered 3D cubes influences their perceived intensity of illumination.

## Method

### Subjects

Twenty subjects participated in the experiments (13 male; mean age = 29.9 years). Subjects were strongly right-handed. Mean laterality scores (Oldfield, 1971) were 70.1 (male) and 92.9 (female). All subjects possessed normal stereo vision, and normal or corrected-to-normal visual acuity.

### Instrumentation

Stimuli were presented on a 23″ RMW9V Dell Alienware LCD monitor (AW2310) using an NVIDIA 3D Vision Pro System video card. Screen resolution was 1920 × 1080 pixels. Frame rate was 120 Hz. Stereo images were realized using polarized shutter glasses and frame-interleave (60 Hz monocular). At a viewing distance of 67 cm the screen dimensions were 41.52° × 24.10°.

### Stimuli

Individual cubes were modeled and rendered using Blender (www.blender.org). Within the modeled Blender environment each cube edge measured 2 m in length. Two virtual cameras positioned 6 cm apart imaged the cubes from a virtual distance of 10 m, at an elevation of 35.4°, to produce stereo pairs. Cubes were rotated into nine unique poses, each having an identically shaped triplet of visible faces. Cubes were rendered on a zero intensity background under low intensity ambient illumination (0.05 arbitrary units). Blender illumination intensity units are arbitrary in that they simply specify the ratio of direct to ambient illumination. Directional lighting was between 6–20 times greater than ambient (0.3–1.0 arbitrary units), and its intensity did not falloff with distance. This simulates a light source at infinity, and ensured that cube face luminance would be homogeneous. The variable intensity lighting came from one of four directions: Above-Left (AL: +24.4° elevation; −90° azimuth); Above-Right (AR: +24.4°; +90°); Below-Left (BL: −24.4°; −90°); and Below-Right (BR: −24.4°; +90°).

**Figure 1:**
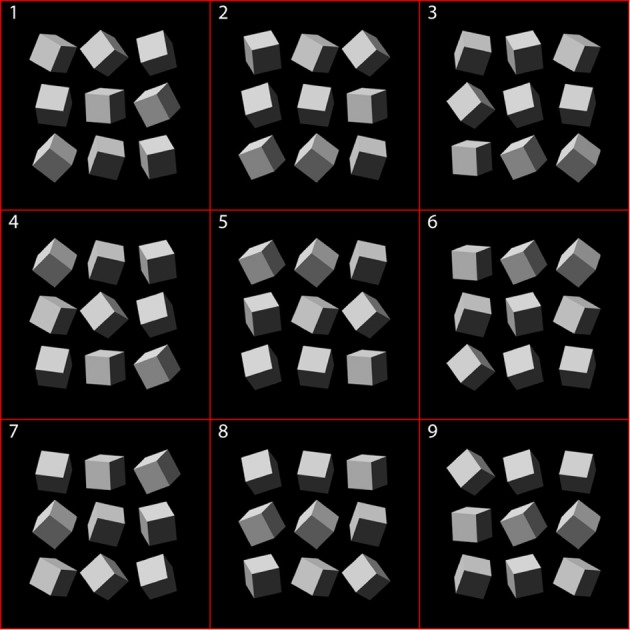
**Cube configurations arranged in the nine unique 3 × 3 arrays**.

**Figure 2:**
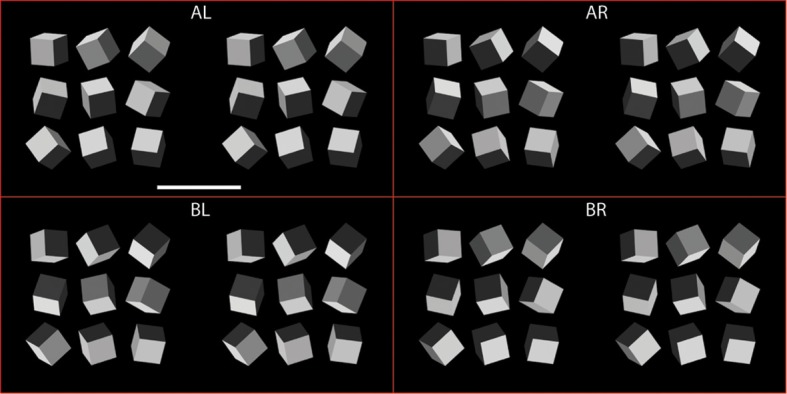
**Four stereo pairs of cube arrays, arranged for crossed fusion, illuminated from each of the four possible directions (AL, Above-Left; BL, Below-Left; AR, Above-Right; BR, Below-Right) at intensity level 15.** Scale bar is 5°.

Individual cubes were positioned in the frontoparallel plane at nine virtual viewing distances ranging from 5.89–13.55 m to create arrays of nine cubes with unique poses. Cube arrays were displayed on the monitor as gamma-corrected 10-bit pseudogray images (Tyler et al., 1992) using MATLAB and Psychtoolbox (Brainard, 1997; Pelli, 1997). The bounding box of individual cubes measured 2.7° × 2.7°. Figure 1 illustrates nine cube configurations arranged in the nine unique 3 × 3 arrays. Which of the nine possible cube configurations was presented on each trial was randomized. Each of these arrays formed one half of a stereo pair. Four stereo pairs of cube arrays are shown in Figure 2 (arranged for crossed fusion), directionally illuminated at the highest intensity level as indicated. The centers of the bounding boxes of the central cubes of each stereo pair array were located ±5.43° from screen center.

**Figure 3:**
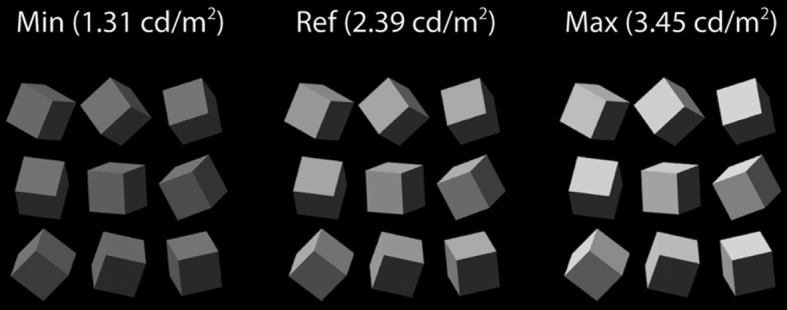
**Three cube arrays virtually illuminated from Above-Left at the minimum, reference, and maximum intensities**.

Cube arrays were illuminated at 15 virtual intensity levels, resulting in mean cube luminances of rendered images ranging in linear steps from 1.31–3.45 cd/m^2^ as viewed through the stereo shutter glasses (5% transmittance). Mean cube luminance under ambient illumination alone was 0.52 cd/m^2^. Figure 3 illustrates three cube arrays virtually illuminated from Above-Left at the minimum, reference, and maximum intensities, corresponding to mean cube luminances of 1.31, 2.39, and 3.45 cd/m^2^, respectively.

### Procedure

Reference cube arrays were always illuminated from Above-Left at the intermediate intensity level, yielding a mean cube array luminance of 2.39 cd/m^2^. Matching cube arrays were virtually illuminated from all four directions at each of the 15 intensity levels. Matching arrays appeared with equal frequency in either the left or right visual field. Matching array visual field and illumination intensity varied quasi-randomly from trial to trial. Subjects were instructed to indicate by left or right button press which of the two cube arrays appeared to be under higher illumination. Using the method of constant stimuli the matching array illumination level required to establish the point of subjective equality (PSE) with the reference array was determined as a function of matching array visual field, illumination elevation, and illumination azimuth. Subjects made a total of 1200 forced-choice judgments about relative array illumination intensity (15 matching array illumination intensities × 2 matching array locations × 4 matching array illumination directions × 10 trials/condition). Psychometric data for each observer in each experimental condition were fit by a two-parameter—PSE and standard deviation (*SD*)—cumulative normal function using a maximum-likelihood criterion. The fitted PSE parameter corresponded to the illumination intensity of the matching array yielding 50% “more intense” relative illumination judgments. Judgments were not speeded and stimuli remained visible until subject response.

## Results

**Figure 4:**
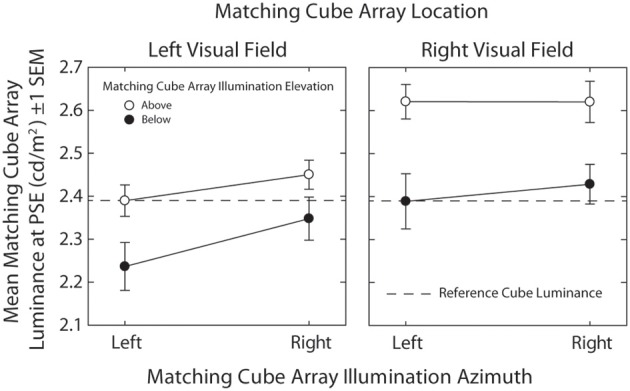
**Mean matching cube array luminance at PSE (±1 s.e.m.) plotted as a function of matching array visual field (Left vs. Right) and illumination azimuth (Left vs. Right), with matching array illumination elevation (Above vs. Below) shown as a parameter**.

Figure 4 plots mean matching cube array luminance at the PSE (±1 s.e.m.) as a function of matching array visual field (Left vs. Right) and illumination azimuth (Left vs. Right), with matching array illumination elevation (Above vs. Below) shown as a parameter. The dashed line indicates mean reference array luminance. Inferential statistical analyses were performed using a 2 (Visual Field: Left vs. Right) × 2 (Illumination Elevation: Above vs. Below) × 2 (Illumination Azimuth: Left vs. Right) within-subjects ANOVA.

There was a significant main effect of visual field [*F*_(1, 19)_ = 6.85, *p* = 0.017, η^2^ = 0.265] where matching cube arrays appeared significantly more intensely illuminated than the reference cube array when they were situated in the left visual field. There was a significant main effect of illumination azimuth [*F*_(1, 19)_ = 16.94, *p* = 0.001, η^2^ = 0.471] where matching cube arrays appeared significantly more intensely illuminated than the reference cube array when they were illuminated from the left. There was a significant main effect of illumination elevation [*F*_(1, 19)_ = 14.47, *p* = 0.001, η^2^ = 0.432] where matching cube arrays appeared significantly more intensely illuminated than the reference cube array when they were illuminated from below. Finally, there was a significant interaction between visual field and illumination azimuth [*F*_(1, 19)_ = 4.74, *p* = 0.042, η^2^ = 0.200] such that the effect of matching cube illumination azimuth was significant for matching arrays situated in the LVF [*t*_(19)_ = 4.31, *p* < 0.001], but not the RVF [*t*_(19)_ = 0.99, *p* = 0.337].

## Discussion

### Effect of visual field

Cube arrays situated in the left visual field appear significantly more intensely illuminated than identical arrays in the right visual field. This result reprises numerous studies showing that many object properties (e.g., perceived intensity, numerosity, size) are exaggerated when situated in the left vs. right visual field (Bowers and Heilman, 1980; McCourt and Jewell, 1999; Nicholls et al., 1999; Jewell and McCourt, 2000; McCourt and Garlinghouse, 2000; Foxe et al., 2003; Charles et al., 2007). As noted previously, the commonly accepted explanation for such leftward attentional biases is that they are a corollary of the right hemisphere's specialization for the deployment of spatial attention, which results in an asymmetric allocation of visuospatial attention favoring left hemispace.

### Effect of illumination azimuth

Cube arrays lit from the left appear more intensely illuminated than identical arrays lit from the right. Ecological explanations for this left-right asymmetry based on image- or scene-based statistics are untenable because light source azimuth, unlike elevation, is not inherently anisotropic. However, since leftward attentional biases have been demonstrated to occur in both space-based (egocentric) and object-based (allocentric) frames of reference (Reuter-Lorenz et al., 1996; Nicholls et al., 2004; Orr and Nicholls, 2005; Pia et al., 2010; Theeuwes et al., 2013), the finding that left-lit cube arrays appear more intensely illuminated than their right-lit counterparts is likely due to the asymmetric (left-biased) distribution of object-based (allocentric) visuospatial attention (Foxe et al., 2003; Orr and Nicholls, 2005; Pia et al., 2010; Chen, 2012). According to this explanation the left halves of individual cubes are more strongly attended than their right halves (allocentric pseudoneglect), making cubes illuminated from the left, whose left halves are more intensely illuminated, appear more intensely illuminated overall than their right-illuminated counterparts. A closely related left-biased brightness effect has been demonstrated with simple two-dimensional objects in the grayscales task (Nicholls et al., 1999), where two mirror image luminance ramps are arranged one above the other. When asked to identify which ramp is overall darker (or brighter), subjects consistently select the ramp whose relevant feature (dark or bright) is on the left. Also strengthening this attentional interpretation is the fact that patients with visuospatial hemineglect, who are densely inattentive to left space or to the left halves of objects, fail to exhibit the normal light-from-left bias (de Montalembert et al., 2010).

### Effect of illumination elevation

Cube arrays lit from below appear more intensely illuminated than identical arrays lit from above. Since scene illumination is ubiquitously from above, one explanation for this effect is that long-term adaptation to light-from-above might result in a reduced sensitivity or responsiveness in mechanisms “tuned” to downward vs. upward illumination, similar to the effects of long-term adaptation on color (Neitz et al., 2002; Delahunt et al., 2004) or contrast perception (Kwon et al., 2009; Zhang et al., 2009). It is noteworthy that this explanation implies the existence of channels selectively tuned to illumination direction. A neurophysiological correlate of such channels might be the subpopulation of neurons in macaque V4 identified by Hanazawa and Komatsu (2001) that respond selectively to luminance-gradient stimuli of various orientations, with a majority of cells tuned to top-bright gradients, consistent with downward illumination of convex objects. Further evidence that channels tuned to light direction might exist is the finding that the light-from-above bias can be modified by experience (Adams et al., 2004).

Our result that bottom-lit objects appear more intensely illuminated than their top-lit counterparts may also be related to the finding that discs with top-dark luminance gradients, seen as concavities due to the light-from-above assumption, are perceived to possess higher contrast than top-bright stimuli, which are perceived as convexities (Chacon, 2004). Despite appearing concave, disks with top-dark luminance gradients are nonetheless consistent with being convexities lit from below. The visual system exhibits a bias to perceive convexity (Sun and Perona, 1997; Langer and Bulthoff, 2001; Champion and Adams, 2007), so while the light-from-above bias apparently outweighs the convexity interpretation in such simple disc stimuli, the fact that top-dark stimuli are consistent with the interpretation of convex objects lit from below might nonetheless confer on these stimuli their greater perceived contrast.

### Effect of lighting direction on perceived intensity and preference

A large majority of photographs and portrait paintings have been discovered to depict scenes in which illumination comes from above—which is not surprising—and from the left, the reasons for which are far less obvious (Sun and Perona, 1998; McManus et al., 2004; Thomas et al., 2008). If lighting preference is equated with the frequency of its representation then by this convention there is a partial dissociation between the effect of lighting direction on perceived intensity and preference. That is, we find that the perceived intensity of illumination is greatest for light from the left, which corresponds with preference as defined by prevalence, but is greatest for light from below, opposite to the preferred direction.

Directional preference has, however, also been defined in terms of visual search efficiency. As mentioned earlier, search is relatively efficient for below/right-lit targets amidst top/left-lit distractors, but is inefficient for the opposite arrangement (Enns and Rensink, 1990; Sun and Perona, 1996a,b, 1998). Sun and Perona (1998) refer to the efficient search condition as revealing a “preference” for light from above, but the efficiently detected target is nonetheless the one lit from below. This search asymmetry suggests that lit-from-below targets may be afforded a special status as featural cues. Perhaps the featural element which affords perceptual pop-out is precisely the greater brightness and/or contrast conferred by being lit from below.

### The relationship between brightness, lightness and perceived illumination

The visual system does not have direct access to either reflectance (R) or illumination (I) but only to their product which is the luminance (intensity) distribution falling on the photoreceptor array: L*(x,y)* = I*(x,y)* • R*(x,y)*. The independent recovery of surface reflectance R *(x,y)* and illumination I*(x,y)* is thus an ill-posed (inverse) problem in that there are innumerable combinations of these two variables that can give rise to any particular intensity distribution, and in the absence of additional information it is impossible to uniquely recover the physically correct solution. As a result of the inverse problem, perceived illumination can only be determined or estimated (correctly or incorrectly) based on knowledge or assumptions (conscious or unconscious) about the reflectance of the target surface or object (Blakeslee et al., 2008; Blakeslee and McCourt, 2012). The 3D stereoscopic rendering of the cubes, the assignment of cube-face surface intensities consistent with a particular illumination direction, and the instructions given to subjects (i.e., on each trial pick the more intensely illuminated array), all strongly supported an interpretation of the cubes as homogeneously reflective objects under illumination of variable intensity and direction. Accordingly, while we have discussed our results in terms of the “perceived intensity of illumination,” it should be noted that under these conditions the relative illumination of the cubes is equivalent to their average relative brightness (perceived intensity).

### Conflict of interest statement

The authors declare that the research was conducted in the absence of any commercial or financial relationships that could be construed as a potential conflict of interest.
